# Physical integrity and residual bio-efficacy of PBO-pyrethroid synergist-treated and pyrethroid-only LLINs after 1.5 years of field use in Western Kenya

**DOI:** 10.1371/journal.pone.0330177

**Published:** 2025-08-12

**Authors:** Job Oyweri, Patrick O. Onyango, Maxwell G. Machani, Josephat Bungei, Yaw A. Afrane, Ming-Chieh Lee, Daibin Zhong, Guofa Zhou, Harrysone Atieli, John Githure, Guiyun Yan

**Affiliations:** 1 Department of Zoology, Maseno University, Kisumu Kenya; 2 International Centre of Excellence for Malaria Research, Tom Mboya University, Homabay, Kenya; 3 Centre for Global Health Research, Kenya Medical Research Institute, Kisumu, Kenya; 4 Department of Medical Microbiology, University of Ghana Medical School, Accra, Ghana; 5 Department of Population Health and Diseases Prevention, University of California, Irvine, California, United States of America; World Health Organization, Regional Office for South-East Asia, INDIA

## Abstract

**Background:**

Long-lasting insecticidal nets (LLINs) are vital for malaria control in sub-Saharan Africa, but their durability is challenged by fabric decay and pyrethroid resistance. This study assessed the physical integrity and bioefficacy of piperonyl butoxide-LLINs (PBO-LLINs) and pyrethroid-only LLINs (pyrethroid-LLINs) after 1.5 years of use in western Kenya, where resistance is widespread.

**Methods:**

A survey on net integrity and insecticide efficacy was conducted in randomly selected households (101–107 per group per visit) from three villages per net type group in Muhoroni Sub-County, Kisumu County. Physical integrity surveys were done after every six months while residual bio-efficacy was after every three months for 18 months. Physical integrity and residual bio-efficacy studies were conducted following WHO guidelines.

**Results:**

PBO-LLINs exhibited higher physical integrity than pyrethroid-LLINs over time. At 18 months, 45.2% (61/135) of pyrethroid-LLINs and 21.8% (31/142) of PBO-LLINs were torn, with pHI values of 2494.1 ± 1696.4 and 1618.6 ± 1056.7, respectively. Net type, net age and house wall structures significantly influenced net integrity (p < 0.05). Torn nets were significantly more common in pyrethroid-LLIN households with mud-unplastered [OR=5.323 (95% CI = 1.685–16.816), p = 0.004] and corrugated iron walls [OR=6.31 (95% CI = 2.10–18.93), p < 0.001] and in PBO-LLIN households with mud-unplastered walls [OR=9.823 (95% CI = 1.487–64.898), p = 0.018]. Against the Kisumu susceptible *Anopheles gambiae* s.s, both net types decreased in mortality at baseline (when new) from 97.6% to 18.4% and 98.6% to 18.5% for pyrethroid and PBO-LLINs respectively at 18 months. Against a Bungoma pyrethroid-resistant *Anopheles gambiae* s.s*,* mosquito mortality with pyrethroid-LLINs declined from 36.9% when new to 6.8% at 18 months, while PBO-LLINs dropped from 55.6% to 11.8%.

**Conclusion:**

Both physical integrity and bioefficacy of LLINs declined significantly within 18 months. The findings demonstrate that not all nets in the field offer maximum protection by this time point, calling for net care education and further evaluation of PBO-LLINs especially in pyrethroid-resistant regions.

## Introduction

Despite widespread pyrethroid resistance, especially in Africa, the use of pyrethroid-LLINs is the primary vector control method. However, this growing resistance has raised concerns about their long-term effectiveness in malaria prevention [1]. In western Kenya, pyrethroid-resistant malaria vectors have been identified, necessitating more effective interventions, while in Benin, such mosquitoes have demonstrated the ability to partially breach compromised LLINs [2–5].

Between 2000 and 2022, the use of LLINs had contributed to about 55.5% drop in malaria deaths in sub-Saharan Africa [6]. Long-lasting insecticidal nets physically act as a barrier to mosquito bites, knock down and kill them depending on their susceptibility [7,8]. These nets have a lifespan of three years but may continue to offer protection beyond this period if the fabric remains intact [9]. In addition to physical protection, the fabric also provides a functional surface area for the insecticides [10,11]. However, studies have reported a loss of net efficacy before the three-year mark due to both fabric deterioration and insecticidal decay, highlighting the need for continuous monitoring of these products across different regions to inform timely replacements and optimize protection [12–14].

According to the Kenya Demographic and Health Survey, 2022, Kenya recorded 54.2% LLINs ownership and a universal coverage of 37.1%. In Western Kenya, Kisumu county in particular, 76% of households owned at least one net and a 47% universal coverage [15]. The statistics suggest that, despite the national distribution efforts, there could be underlying factors such as people’s perceptions, net condition or certain behaviours that lead to people losing their nets affecting ownership and usage [16–19].

Physical integrity of nets is key in malaria control [20]. Over time, nets deteriorate gradually especially in rural areas where usage tends to be high [13]. The development of holes on nets increases human-vector interaction, increasing malaria transmission risk [16,21–23]. People’s perceptions on the effectiveness of damaged or torn nets, lead to their abandonment, affecting ownership and usage [24,25]. Thus, evaluating the effectiveness of LLINs without considering their physical integrity risks misinterpreting the results.

The WHO has recommended the use of piperonyl butoxide long lasting nets (PBO-LLINs) to mitigate pyrethroid resistance [9]. Evidence from Nigeria [26] and Uganda [27] demonstrates their effectiveness in reducing malaria transmission. These nets have been distributed in Western Kenya (a region characterized by high malaria transmission and significant pyrethroid resistance) to supplement the existing pyrethroid-LLINs [28]. Given the potential for global distribution of PBO-LLINs, extensive fabric integrity and bioefficacy are essential to understand their performance under field conditions. This study aimed to assess the physical integrity and bioefficacy of PBO-LLINs compared to pyrethroid-LLINs under field conditions in Western Kenya.

## Methods

### Study area

The study was conducted in Muhoroni Sub-County, Kisumu County, Western Kenya. The area occupies 425.3 km² with 154,116 residents according to the 2019 national census [29]. Sugarcane and rice farming are the main economic activities benefitting from the region’s hot and wet climate [30–32]. The main vectors responsible for malaria endemicity in Western Kenya are *Anopheles gambiae* sensu stricto (s.s), *Anopheles funestus* sensu lato (s.l.) and *Anopheles arabiensis* [32–35].

### Study design

Repeated cross-sectional study design was used to conduct physical integrity and bioefficacy surveys. Net integrity survey and bio-efficacy studies were conducted semi-annually and quarterly for 18 months from January 2022 to July 2023 post-mass net distribution. The nets were distributed in December 2021 by International center of excellence for malaria research (ICEMR), Homabay. The nets were uniquely marked with an abbreviation “ICEMR”, followed by the code assigned to the household to distinguish them from non-study nets. The surveys were conducted in two groups; PBO-LLINs (PermaNet 3.0) and pyrethroid-LLIN (PermaNet 2.0), both from Vestergaard, Netherlands. The pyrethroid-LLIN is made of 100% polyester [36], whereas the PBO-LLIN consists of a 100% polyethylene roof and 100% polyester sides [37]. During each visit, different households were surveyed.

### Sample size calculation

Sample size was calculated using Cochran’s formula [38]:


$$S=\frac{Z^{2}P(1-P)}{d^{2}}\;\to \frac{1.96^{2}\times 0.76\times 0.23}{0.05^{2}}\;=\;268.6$$

Where; S = Infinite sample size, Z = Z-score at 95% confidence level (1.96), P = percentage of households owning at least one net in Kisumu county according to the Kenya Demographic and Health Survey in 2022, (0.76) [15], d = Precision 0.05.

Application of finite correction factor formula:


$$\text{Sample\textbackslash{} size\textbackslash{} (n)}=\;\frac{S}{1+\left(\frac{s-1}{N}\right)}=\frac{268.6}{\;1+(\frac{268.6-1}{155})}\;=\;98.5$$

Where; N refers to the number of households per group nets. Thus, at least 98 households were suitable for the study.

### Fabric integrity survey

#### Households, population and nets surveyed across time points.

The number of households, population and the nets surveyed at different time points are summarized in table 1 below. All available study nets were surveyed.

**Table 1:** Households, population and nets surveyed across time points.

Time (months)	Type of net	Households	Residents	Nets surveyed
6	Pyrethroid-LLIN	101	231	124
	PBO-LLIN	104	316	169
12	Pyrethroid-LLIN	102	214	116
	PBO-LLIN	104	276	137
18	Pyrethroid-LLIN	107	282	135
	PBO-LLIN	106	257	142

#### Potential factors that may contribute to reduced fabric integrity.

The majority of the house walls were plastered or coated with mud (>50%) and had iron corrugated sheet roofs with open eaves. A research assistant accompanied by a community health promoter (CHP), recorded house coordinates, household population, number of nets owned, net usage (previous night net usage), sleeping patterns by age groups, household structure, net type and net age into open data kit (ODK). The CHPs had a master list having households’ population and the number of nets available. To discourage non-use of nets due to their storage as demonstrated in other studies [39,40], the CHPs conducted bi-weekly house surveillance on net use and sensitizing the community on the importance of LLINs in malaria control. Households that consistently did not use their nets, were excluded from the study.

#### Fabric integrity measurements.

**Fig 1:**
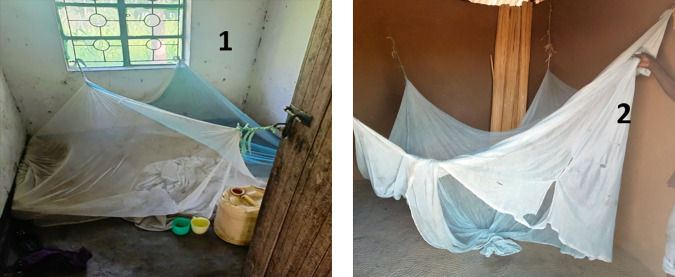
A good net tucked under a mattress on the floor strained to window grills and hand forged door metal lock; 2 - A torn net offering no maximum protection.

Following the WHO guidelines [41], net hole counts from seams, roof panel, upper side and lower side were recorded. The roof was defined as the topmost part of the net. The upper side was defined as the upper half of the net just below the net roof while the lower side was the bottom half closer to the bed or sleeping space. Holes were categorized as follows: size 1 (0.5–1.99 cm) smaller than a thumb, size 2 (2–9.99 cm) in between a thumb and closed fist, size 3 (10–24.99 cm) in between fist and head and size 4 (≥25 cm) bigger than a head. To categorize fabric net condition (Fig 1), the Proportional Hole Index (pHI) for each net was calculated based on the number of holes in each category multiplied by the index attributed to that size: Size 1 holes × 1, Size 2 holes × 23, Size 3 holes × 196, and Size 4 holes × 576 [41].

#### LLINs bio-efficacy.

Every three months after net distribution, two nets were collected from each house wall structure (mud-plastered, exposed mud otherwise referred as mud-unplastered, and brick/cement/block) in each LLIN group (PBO-LLIN or pyrethroid-LLIN), totalling six nets per group. New nets were issued to houses from which they were collected. Bio-efficacy studies were conducted using standard WHO cone bioassays.

Five 25 cm x 25 cm pieces of net material were cut from the roof and four sides, wrapped in aluminium foil and stored at 4^o^C before WHO cone assays. One hundred non-blood fed, 3–6-day old female Bungoma-resistant *Anopheles gambiae* s.s strain [42] were used to evaluate bioefficacy of the net. Each side of the net sample (one piece) was tested using four cones, each containing five mosquitoes (total 20 mosquitoes per side). After 3 minutes of exposure, mosquitoes from each side were transferred into separately labelled paper cups (one cup per side) and monitored for knockdown at 30-minute intervals for 1 hour, and mortality after 24 hours. Twenty mosquitoes per net side were also tested against the positive (new and unused nets) and negative (nets without insecticides) controls. During the 24h monitoring, the mosquitoes were fed on 10% sugar solution. The bioassay was repeated if over 20% mortality in the negative control was observed. Same procedure was repeated with the non-blood fed female lab-reared Kisumu susceptible *Anopheles gambiae* s.s strain mosquitoes [43]. The bioassays were conducted under controlled environmental conditions consistent with WHO guidelines at 27 ± 2 °C and 80 ± 10% relative humidity. Trained personnel conducted the tests, mosquitoes were handled with care to minimize stress and uniformity across tests was ensured (i.e., cups, cones, cotton, sugar solution etc). The bioassays were conducted in Kenya Medical Research Institute, Kisumu, Kenya at the Centre for Global Health Research (CGHR).

#### Ethics approval and consent to participate.

Maseno University Scientific and Ethics Review Committee (MUSEREC) approved this research under study number MUERC/00778/19. The Ministry of Health, Kisumu, Kenya provided authorization of carrying out the research in the community. From 1^st^ to 9^th^ December 2021, adults willingly consented and assented for young children (written informed consent). Study participants could withdraw from the study at any time.

#### Inclusivity in global research.

Further information to the ethical, cultural and scientific aspects specific to inclusivity in global research has been provided in the Supporting Information (SX Checklist).

### Data analysis

All statistical analyses were performed using IBM SPSS software version 27. The normality of continuous variables was assessed using the Kolmogorov-Smirnov test [44]. Where significant deviations from normality were observed, appropriate non-parametric methods were employed. The Proportional Hole Index (pHI) for each net was computed by applying WHO-defined weights to the number of holes within each size category. Nets were classified as ‘good’ (pHI ≤ 64), ‘moderately damaged’ (pHI between 65 and 642), or ‘Torn’ (pHI > 642), following WHO criteria [41]. Net bioefficacy was assessed via WHO cone bioassays, based on knockdown and mortality outcomes. Nets were categorized as ‘Optimal’ (≥80% mortality or ≥95% knockdown), ‘Minimal’ (≥50% mortality or ≥75% knockdown), or ‘Not Effective’ (<50% mortality or <75% knockdown) [45]. Differences in net condition across time points and intervention arms (PBO-LLIN vs. pyrethroid-LLIN) were examined using Pearson’s chi-square test. Assumptions regarding the independence of observations and adequacy of expected cell counts were verified and satisfied. To identify predictors of net condition, a multinomial logistic regression model was fitted with net condition as the outcome variable. Multicollinearity assumptions were tested and verified. Statistical significance was set at p ≤ 0.05. Where applicable, 95% confidence intervals were reported alongside point estimates.

## Results

### Characteristics of the households surveyed

**Fig 2:**
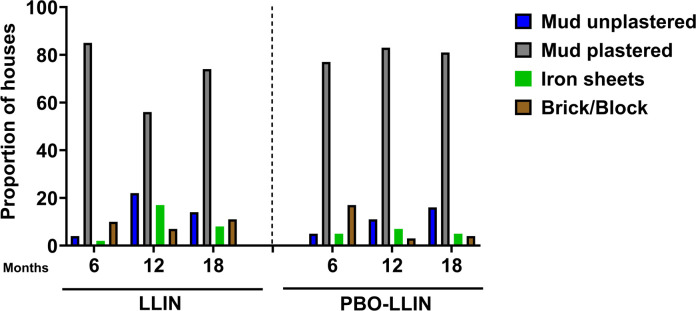
Distribution of houses by wall structures (LLIN denotes Pyrethroid-only LLIN).

Of the houses surveyed at each time point for net integrity, the majority were made of mud plastered walls (>54.9%). At six months, 84.2% (85/101) of houses in the pyrethroid-LLIN group and 74.0% (77/104) in the PBO-LLIN group were mud-plastered. At 12 months, there were 54.9% (56/102) against 79.8% (83/104) mud plastered houses respectively. At the 18^th^ month survey, 69.2% (74/107) households in pyrethroid-LLIN group had mud-plastered walls compared to 76.4% (81/106) as shown in fig 2.

In the two groups, ≥ 96.3% of the nets available in the households were used the previous night prior to the survey. Specifically, at 6 months 99.2% (123/124) of pyrethroid-LLINs and 98.8% (167/169) of PBO-LLINs were reported to be used the night before the survey. At 12 months, the usage rates slightly decreased to 97.4% (113/116) for pyrethroid-LLINs and 97.1% (133/137) for PBO-LLINs. By 18 months, the number of nets being used the night prior to the survey was still high, with 96.3% (130/135) for pyrethroid-LLINs and 96.5% (137/142) for PBO-LLINs. The usage of the available nets did not differ significantly throughout the study period (χ^2^ = 4.191, df = 2, p = 0.123).

### Net integrity over time in PBO and Pyrethroid-LLIN groups

A decline in net integrity was observed over time with PBO-treated nets consistently performing better than pyrethroid-LLINs (Table 2). At 6 months, the majority of PBO nets were in good condition; 98.22% (166/169) compared to 68.55% (85/124) in the pyrethroid-LLIN group. By 12 months, the proportion of nets in good condition dropped especially for pyrethroid-LLINs, 37.07% (43/116) compared to 78.83% (108/137) of the nets in the PBO group. At 18 months, 59.15% (84/142) in the PBO arm and 28.89% (39/135) (Table 2) in the pyrethroid-LLIN arm were in good condition. The respective pHIs for the net conditions are captured in Table 3. The remainder of the nets were either moderately damaged or torn reducing their protection. Overall, there was significant differences in net integrity at each time point across the two groups (p < 0.001); (Table 2). The fabric integrity performance of the two nets at 6 months versus 12 months versus 18 months was assessed. There was significant difference in fabric integrity at 6 months versus 12 months between the individual net types; pyrethroid-LLINs (χ^2^ = 45.164, df = 2, p < 0.001) and PBO-LLINs (χ^2^ = 30.767, df = 2, p < 0.001). Significant differences in fabric integrity were also recorded at 6 months versus 18 months in pyrethroid-only LLINs (χ^2^ = 57.654, df = 2, p < 0.001) and PBO-LLINs (χ^2^ = 75.320, df = 2, p < 0.001). Considering 12 months vs 18 months, the pyrethroid-only LLINs recorded no significant difference in fabric integrity (χ^2^ = 2.343, df = 2, p = 0.310) while PBO-LLINs showed significant difference (χ^2^ = 17.230, df = 2, p < 0.001).

**Table 2:** Pyrethroid LLIN versus PBO-LLIN fabric integrity at each time point.

Intervention		Pyrethroid-LLIN (N = 124) Frequency (%)	PBO-LLIN (N = 169) Frequency (%)	Pyrethroid-LLIN (N = 116) Frequency (%)	PBO-LLIN (N = 137) Frequency (%)	Pyrethroid-LLIN (N = 135) Frequency (%)	PBO-LLIN (N = 142) Frequency (%)
Age		6 months	6 months	12 months	12 months	18 months	18 months
Net status	Good	85 (68.55)	166 (98.22)	43 (37.07)	108 (78.83)	39 (28.89)	84 (59.15)
	Moderately damaged	31 (25)	3 (1.78)	23 (19.83)	21 (15.33)	35 (25.93)	27 (19.01)
	Torn	8 (6.45)	0	50 (43.10)	8 (5.84)	61 (45.19)	31 (21.83)
*P-*value		χ^2^ = 51.502, df = 2, p < 0.001		χ^2^ = 57.135, df = 2, p < 0.001		χ^2^ = 27.119, df = 2, p < 0.001	

**Table 3:** pHI for net status.

Intervention		Pyrethroid-LLIN (N = 124) pHI ± SD	PBO-LLIN (N = 169) pHI ± SD	Pyrethroid-LLIN (N = 116) pHI ± SD	PBO-LLIN (N = 137) pHI ± SD	Pyrethroid-LLIN (N = 135) pHI ± SD	PBO-LLIN (N = 142) pHI ± SD
Age		6 months	6 months	12 months	12 months	18 months	18 months
Status	Good	11 ± 17.2	0.5 ± 3.2	13.3 ± 21.2	5.8 ± 12.9	14.8 ± 18	8.4 ± 15.2
	Moderately damaged	237 ± 160.2	154.3 ± 72.2	129.7 ± 63.7	277.3 ± 187	354.7 ± 166.2	280.7 ± 203.7
	Torn	1650.3 ± 1025.1		4124.2 ± 1873	1825 ± 1378.5	2494.1 ± 1696.4	1618.6 ± 1056.7

### Position of holes on LLINS

Across time points, pyrethroid-LLINs showed higher hole rates on the upper and roof sides while PBO-LLINs exhibited higher rates on the lower side of the net. Overall, across time points, there were significant differences in mean position holes across interventions with pyrethroid-LLINs showing higher hole counts than PBO-LLINs (p < 0.008); Table 4.

**Table 4:** LLINs hole positions over time.

Net Age	Hole position	Intervention	Nets	Hole counts, n/N (%)	*P*-Value
6 months	Roof	Pyrethroid-LLIN	124	286/844 (33.89)	<0.001
		PBO-LLIN	169	2/27 (7.41)	
	Upper side	Pyrethroid-LLIN	124	260/844 (30.81)	<0.001
			PBO-LLIN	169	4/27 (14.81)
	Lower side	Pyrethroid-LLIN	124	181/844 (21.45)	<0.001
			PBO-LLIN	169	15/27 (55.56)
	Seams	Pyrethroid-LLIN	124	117/844 (13.86)	<0.001
			PBO-LLIN	169	6/27 (22.22)
12 months	Roof	Pyrethroid-LLIN	116	623/1723 (36.16)	<0.001
		PBO-LLIN	137	83/237 (35.02)	
	Upper side	Pyrethroid-LLIN	116	554/1723 (32.15)	<0.001
			PBO-LLIN	137	60/237 (25.32)
	Lower side	Pyrethroid-LLIN	116	338/1723 (19.62)	<0.001
			PBO-LLIN	137	83/237 (35.02)
	Seams	Pyrethroid-LLIN	116	208/1723 (12.07)	<0.001
			PBO-LLIN	137	11/237 (4.64)
18 months	Roof	Pyrethroid-LLIN	135	355/1362 (26.06)	<0.001
		PBO-LLIN	142	130/694 (18.73)	
	Upper side	Pyrethroid-LLIN	135	399/1362 (29.30)	<0.001
			PBO-LLIN	142	209/694 (30.12)
	Lower side	Pyrethroid-LLIN	135	389/1362 (28.56)	0.008
			PBO-LLIN	142	300/694 (43.23)
	Seams	Pyrethroid-LLIN	135	219/1362 (16.08)	<0.001
			PBO-LLIN	142	55/694 (7.93)

N refers to the total number of holes in the net, n represents the number of holes at the particular side. The Mann-Whitney U test was used to compare the differences in hole counts between pyrethroid-only LLIN and PBO-LLIN nets at 6, 12, and 18 months, across different hole positions (roof, upper side, lower side, seams).

### Factors associated with net integrity

**Fig 3:**
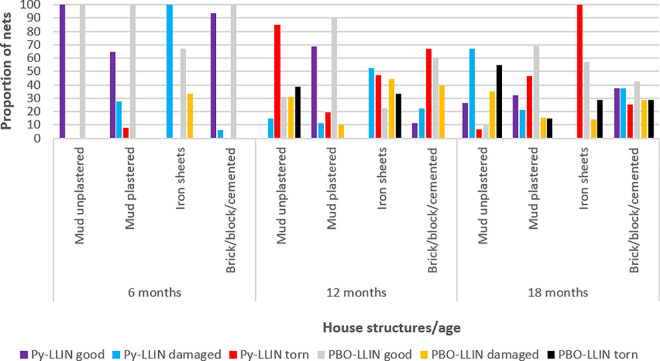
Net status in regard to type of housing. Damaged represents moderately damaged, Py-LLIN represents standard LLIN.

A multinomial logistic regression predicted net age, net type, net usage and house wall structure to be significantly associated with net integrity (Table 5). The likelihood of a net being moderately damaged increased by 13.8% [OR=1.1388 (95% CI = 1.089–1.189), P < 0.001] with increase in age. Pyrethroid-LLINs were 4.8 times more likely to be moderately damaged compared to PBO-LLINs [OR=4.8 (95% CI = 3.099–7.441), P < 0.001]. Households (Fig 3) in mud unplastered wall houses were 4.4 times more likely to have moderately damaged nets [OR=4.421 (95% CI=(1.774–11.017), P = 0.001] while those in corrugated iron walls were 8.8 times more likely [OR=8.771 (95% CI=(3.095–24.856), P < 0.001], when compared to cemented/brick/block wall households. Considering torn nets, the likelihood of a net being torn increased by 29% [OR=1.29 (95% CI = 1.222–1.361), P < 0.001] with increase in age. Pyrethroid-LLINs were 10 times more likely to be torn compared to PBO-LLINs [OR=10.013 (95% CI = 6.101–16.436), P < 0.001]. Households in mud unplastered house walls were 8.6 times more likely to have torn nets [OR=8.601 (95% CI = 3.214–23.015), P < 0.001] while those in corrugated iron walls were 14.8 times more likely [OR=14.786 (95% CI = 4.772–45.81), P < 0.001] when compared to cemented/brick/block wall households.

**Table 5:** Factors associated with mosquito net integrity.

Net physical condition	Variable	Details	Coefficient	Odd ratio (95% CI)	*P-Value*
Moderately damaged	Net Age	Age in months	0.129	1.138 (1.089-1.189)	<0.001
	Sleeping pattern	Age 0–4	0.125	1.134 (0.675-1.904)	0.635
		Age 5–14	−0.179	0.836 (0.416-1.680)	0.616
		Age ≥ 15	−0.092	0.912 (0.437-1.906)	0.807
	Net type	Pyrethroid-LLIN	1.569	4.802 (3.099-7.441)	<0.001
		PBO-LLIN	0^b^		
	Net usage	No	−0.742	0.476 (0.083-2.721)	0.404
		Yes	0^b^		
	Type of house wall	Mud unplastered	1.486	4.421 (1.774-11.017)	0.001
		Mud plastered	−0.315	0.730 (0.356-1.498)	0.391
		Corrugated iron house	2.171	8.771 (3.095-24.856)	<0.001
		Brick/Cement/stone	0^b^		
	Population	Population under net	0.341	1.406 (0.689-2.870)	0.349
Torn	Net Age	Age in months	0.254	1.290 (1.222-1.361)	<0.001
	Sleeping pattern	Age 0–4	−0.544	0.580 (0.171-1.972)	0.383
		Age 5–14	−1.011	0.364 (0.110-1.207)	0.098
		Age ≥ 15	0−.772	0.462 (0.137-1.555)	0.212
	Net type	Pyrethroid-LLIN	2.304	10.013 (6.101-16.436)	<0.001
		PBO-LLIN	0^b^		
	Net usage	No	−1.388	0.250 (0.039-1.579)	0.140
		Yes	0^b^		
	Type of house wall	Mud unplastered	2.152	8.601 (3.214-23.015)	<0.001
		Mud plastered	−0.241	0.786 (0.348-1.775)	0.562
		Corrugated iron house	2.694	14.786 (4.772-45.810)	<0.001
		Brick/Cement/stone	0^b^		
	Population	Population under net	0.797	2.218 (0.652-7.550)	0.202

Dependent variable: net status (categorized as “Good,” “moderately damaged,” or “Torn”), net status reference category: Net in “Good” condition. Independent variables: Net age, sleeping pattern by age group, net type, net usage, type of house wall, population. Reference group; 0^b^

### Factors affecting pyrethroid-LLINs integrity

As shown in table 6, a multinomial logistic regression revealed that type of house wall affected pyrethroid-LLIN integrity. Nets in mud unplastered house walls had a 3.6 times higher likelihood of damage [OR=3.599 (95%CI = 1.115–11.619), p = 0.032] and 5.3 times higher likelihood [OR=5.323 (95% CI = 1.685–16.816),p = 0.004] of being torn compared to those in brick/cement/stone walled houses. Nets from corrugated iron walled houses had 4.1 times higher likelihood of damage [OR=4.12 (95% CI = 1.772–9.56), p < 0.001] and 6.3 times higher likelihood of being torn [OR=6.31(95%CI = 2.10–18.93), p < 0.001] when compared with those in brick/cement/stone walled houses. Increase in net age was significantly associated with higher torn rates [OR=1.206 (95%CI = 1.135–1.281), p < 0.001].

**Table 6:** Factors affecting pyrethroid-LLINs integrity.

Net physical condition	Variable	Details	Coefficient	Odd ratio (95% CI)	P value
Moderately damaged	Population	Population under net	0.831	2.296(0.927-5.686)	0.072
	Net age	Age in months	0.054	1.055(0.998-1.117)	0.060
	Sleeping pattern	Age 0–4	0.079	1.083(0.618-1.896)	0.781
		Age 5–14	−0.541	0.582(0.237-1.428)	0.238
		Age ≥ 15	−0.674	0.510(0.189-1.372)	0.182
	Type of house wall	Mud unplastered	1.281	3.599(1.115-11.619)	0.032
		Mud plastered	−0.003	0.997(0.419-2.373)	0.995
		Corrugated iron house	1.415	4.12(1.772-9.56)	<0.001
		Brick/Cement/stone	0^b^		
Torn	Population	Population under net	1.444	4.238(0.949-18.922)	0.058
	Net age	Age in months	0.187	1.206(1.135-1.281)	<0.001
	Sleeping pattern	Age 0–4	−0.955	0.385(0.079-1.882)	0.238
		Age 5–14	−1.626	0.197(0.044-0.879)	0.033
		Age ≥ 15	−1.469	0.230(0.050-1.052)	0.058
	Type of house wall	Mud unplastered	1.672	5.323(1.685-16.816)	0.004
		Mud plastered	0.043	1.044(0.429-2.541)	0.924
		Corrugated iron house	1.842	6.31(2.10-18.93)	<0.001
		Brick/Cement/stone	0^b^		

Dependent variable: net status (categorized as “Good,” “moderately damaged,” or “Torn”), net status reference category: Net in “Good” condition. Independent variables: population, Net age, sleeping pattern by age group, type of house wall. Reference group; 0^b^

### Factors affecting PBO-LLINs integrity

A multinomial logistic regression revealed that the type of house wall affected PBO-LLIN integrity (table 7). Nets in houses with mud unplastered walls had a 9.8 times higher likelihood of being torn [OR = 9.823 (95% CI: 1.487–64.898), p = 0.018] compared to those in brick/cement/stone-walled houses. An increase in net age was significantly associated with higher rates of damage and tearing (p < 0.001).

**Table 7:** Factors affecting PBO-LLINs integrity.

Net physical condition	Variable	Details	Coefficient	Odd ratio (95% CI)	P value
Moderately damaged	Population	Population under net	0.326	1.385 (0.431-4.456)	0.585
	Net age	Age in months	0.240	1.272 (1.172-1.380)	<0.001
	Sleeping pattern	Age 0–4	0.030	1.031 (0.282-3.771)	0.964
		Age 5–14	−0.059	0.943(0.281-3.161)	0.924
		Age ≥ 15	0.205	1.228 (0.369-4.088)	0.738
	Type of house wall	Mud unplastered	1.254	3.504(0.811-15.145)	0.093
		Mud plastered	−1.164	0.312(0.089-1.096)	0.069
		Corrugated iron house	1.520	4.572(0.985-21.229)	0.052
		Brick/Cement/stone	0^b^		
Torn	Population	Population under net	0.521	1.684 (0.297-9.532)	0.556
	Net age	Age in months	0.420	1.522(1.324-1.750)	<0.001
	Sleeping pattern	Age 0–4	0.116	1.123(0.194-6.491)	0.897
		Age 5–14	−0.280	0.756(0.130-4.399)	0.755
		Age ≥ 15	−0.032	0.968(0.169-5.559)	0.971
	Type of house wall	Mud unplastered	2.285	9.823(1.487-64.898)	0.018
		Mud plastered	−1.228	0.293(0.051-1.667)	0.166
		Corrugated iron house	1.922	6.832(0.842-55.448)	0.072
		Brick/Cement/stone	0^b^		

Dependent variable: net status (categorized as “Good,” “moderately damaged,” or “Torn”), net status reference category: Net in “Good” condition. Independent variables: population, Net age, sleeping pattern by age group, type of house wall. Reference group; 0^b^

### Net bioefficacy result

**Fig 4:**
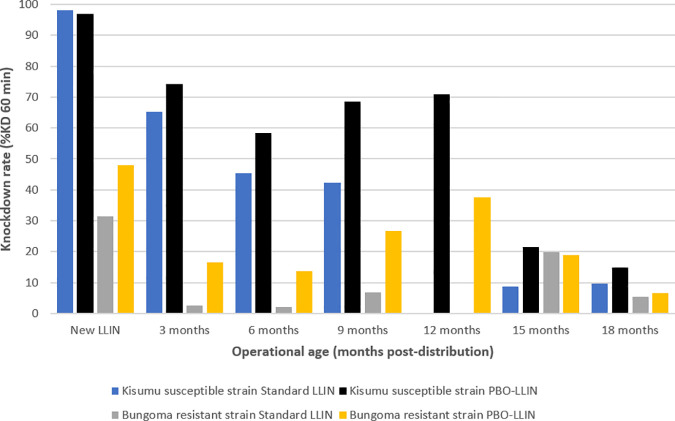
LLINs knockdown performance over time.

As shown in Fig 4, the bioefficacy testing of pyrethroid-LLINs against the Kisumu susceptible strain showed a progressive decline in knockdown rates, from 98% at baseline to 45.5% at month 6 and 9.8% at month 18. In contrast, PBO-LLINs initially dropped from 96.9% at baseline to 58.5% at month 6, increased to 71% at month 12, before sharply declining to 14.8% at month 18. At baseline, 6 and 18 months, there was no significant (p > 0.05) difference in knockdown rates between the PBO-LLIN and pyrethroid-LLIN group.

When tested against the Bungoma pyrethroid-resistant strain, the knockdown rates of pyrethroid-only nets declined from 31.4% at baseline to 2.2% at month 6 and 5.4% at month 18. PBO-LLINs also showed a reduction in effectiveness, with knockdown decreasing from 48.1% at baseline to 13.6% at month 6, 37.6% at month 12, and 6.7% at month 18.

**Fig 5:**
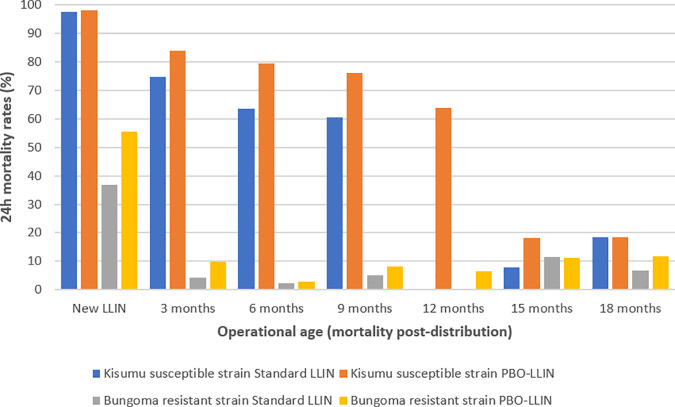
Graph showing LLINs mosquito mortality across time points.

Against the Bungoma resistant strain, when new, the PBO net demonstrated 55.6% mortality against 36.9% for the pyrethroid-LLIN. However, over time, from three months to 18 months, mortality ranged between 2.8% and 11.8% for the PBO-LLIN when compared to pyrethroid-LLIN (2.3% and 11.6%); (fig 5). At baseline, mortality rates significantly differed between the two types of intervention (<p = 0.05) while at 6 and 18 months, mortality rates were not significantly different. The two types of LLINs had higher efficacy against the Kisumu susceptible strain. The PBO-LLIN showed almost similar mortality with pyrethroid-LLIN when new and unused (98.1% vs. 97.6%). The maximum mortality was 83.8% and 74.7% for the PBO-LLIN and pyrethroid-LLIN respectively, demonstrated at three months. Despite PBO-LLIN demonstrating higher mosquito killing rates across time points, at 18 months, the effect was similarly low to that of pyrethroid-LLIN (18.5% vs. 18.4%). 

## Discussion

This study reports a decline in the physical and insecticidal condition of LLINs over an 18-months period. However, PBO-LLINs showed higher physical integrity and bioefficacy than pyrethroids-LLINs. A study conducted in Uganda by Mechan et al. [43] has reported significant deterioration within two years of net use. Similar to other studies in Tanzania [46,47], the current study suggests that not all nets provide maximum protection in the field due to fabric and insecticidal decay.

The pyrethroid-LLIN had higher odds of being moderately damaged or torn than the PBO net. Previous studies have shown that people’s perceptions can influence how they handle health interventions [25,48–50]. While our study did not assess perceptions, it is possible that the distinct two-colour design of the PBO net, with a blue roof panel and white sides, may make it appear special to some residents. This perception coupled with messaging during LLIN distribution [51] highlighting enhanced protection of PBO nets against resistant mosquitoes may have encouraged better PBO net care.

In contrast to Minta et al., [21], the top and roof of pyrethroid-LLINs were more vulnerable to developing holes, which are critical vector entry points [52], potentially compromising their physical protection. Similarly, Feio-dos-Santos et al., [53] observed greater damage on the upper side of rectangular LLINs. In this rural setting, with diverse house structures and varying user behaviors such as snagging on a solid [54], improper installation and strained hanging points [55,56] may have contributed to the observed damage on the top and roof of pyrethroid-LLINs unlike PBO-LLINs that is made of polyethylene material roof and perceived effective necessitating care [57]. Although the current study is limited by lack of this data, anecdotal field observations during household visits suggested variations in net hanging practices across households between the interventions, which may have influenced this pattern of damage. Future studies should consider systematically evaluating net hanging methods to better understand their role in physical integrity outcomes. Consistent with findings by Lukole et al., [47] in Tanzania, the present study observed more holes on the lower side of PBO LLINs. Differences in living conditions and net care variations play a role in net integrity [58]. For instance, careless net tucking, can cause abrasions from bed frames, bolts, or rough surfaces, eventually leading to tears [59]. In some households lacking proper beds [47], alternative sleeping arrangements, such as sleeping on floors or papyrus mats, could increase the risk of net damage on the lower side. These torn rates at different LLINs positions and variation from households highlight the need for targeted community education on proper net handling and care to prolong net physical integrity and maintain effectiveness.

Compromised LLINs fabric integrity has far-reaching implications for malaria control programs, particularly in areas with high pyrethroid resistance. First, there is increased risk of malaria infections due to continuous human-vector contact [60]. Second, perceived infectiveness will lead to net abandonment by households [46,61,62], reducing LLIN coverage [17,63]. These outcomes threaten high-resistance settings, where pyrethroid-LLINs effectiveness is compromised. Therefore, communities have to be enlightened via targeted education programme on ways of properly handling and caring for the nets to prolong lifespan. This approach not only enhances malaria prevention but also reduces operational costs by delaying premature net replacement.

The present study found that nets in mud-unplastered and iron-corrugated-walled houses were more likely to be moderately damaged or torn compared to those in brick or stone, or block houses. Similarly, Mechan et al., [43] reported that nets in traditional houses were 3.4 times more likely to be damaged. Mud-unplastered walls and rusted iron sheet walls often have rough surfaces, which may increase the risk of tears, especially if nets are abruptly yanked off. Additionally, frequent contact of nets and these rough surfaces could contribute to wear and tear over time, especially if nets are not properly handled. Individuals in these house structures may be more likely exposed to malaria infections compared to other house structures affecting entire community. Malaria control programmes should consider providing trainings on net handling practices in these challenging environments.

The majority of available nets were reported to have been used the night before the survey, consistent with findings from other settings [14,64,65]. The high LLIN usage observed in this study area aligns with its malaria endemicity [66]. The bi-weekly CHPs visits must have been a constant reminder of net use in this setting. Consistent net usage in the study area likely played a key role in physical integrity decay. A study by Smith et al., [63] demonstrating the association of net use and physical decay in Siaya, western Kenya (a malaria endemic area) showed that 309/543 (57%) of the nets in use were damaged. A study conducted by Mutuku et al., [13] in Coastal Kenya found net usage to be a stronger predictor of physical integrity with nets in use likely to be damaged. Given that net usage is essential and unavoidable, it is crucial to educate and train communities on proper net care to prolong their effectiveness as a malaria control tool. Ministries of health (MoH) introduced PBO nets to mitigate pyrethroid resistance. Despite the current study showing PBO net outperforming the pyrethroid-LLIN in killing the Bungoma resistant strain at baseline, the net fails to attain mortality threshold of ≥80%. The current study is inconsistent with that of Gichuki et al., [67] who demonstrated that the Olyset^®^ Plus net (permethrin and PBO-treated) passed the WHO mortality threshold (≥80%) against Kisumu susceptible *Anopheles gambiae* strain up to 18 months of use in Kenya. Similarly in Tanzania, Martin et al., [68] demonstrated that over 80% of PBO nets passed the WHO efficacy criteria against Kisumu susceptible *Anopheles gambiae* strain up to three years of net use. The results in the current study suggest limited additional benefits of PBO net in managing pyrethroid resistant vectors. The deterioration in fabric quality may have contributed to insecticidal decay [12,69], although this association was not determined. Additionally, human behaviors such as frequent washing [70,71], net handling [71,72], and overall care [73] may have influenced the findings. These results suggest that operational performance of the PBO nets may be insufficient in a high pyrethroid-resistant area where fabric decay co-occur. Educating communities on proper LLIN care is essential to enhance their effectiveness in malaria control. Furthermore, the results of this study highlight the need for further research on PBO nets to better understand their malaria control potential in regions with high pyrethroid resistance.

Limitations: There were a few limitations associated with this study. These are stated here:

The sources of net holes and factors affecting net bio-efficacy were not determined. Sources of net holes could elaborately inform fabric integrity. Factors affecting net bioefficacy such as washing patterns, hanging patterns, presence of proper beds, airing behaviours, among others were not documented leaving the study to rely on speculations based on existing literature. Collection of this information could supplement interpretation of our results. The use of the available nets was self-reported, lacking no proper verification. We are aware that such self-reported data may be influenced by recall bias or social desirability bias which in turn may have led to overestimation of our results. Similar studies seeking to conduct LLINs evaluations in the context of physical integrity and bioefficacy should consider optimizing ways on verification of net use patterns. We did not collect data on the use of other interventions to control malaria such as the use of mosquito coils, repellents, burning of cow dung among others. These behaviours may have led to underutilization of the nets which in turn affects interpretation of the current study findings. At 12 months, the study experienced mosquito maintenance challenges leading to missing bioefficacy data at the 12^th^ month for pyrethroid-LLINs. Future studies should optimize strategies of maintaining the lab reared and resistant mosquitoes to sustain the bioassays throughout the study period.

## Conclusion

Nets in mud-unplastered and iron-sheet homes were more prone to tears, reducing their protective value. With only 28.9% of pyrethroid-LLINs and 59.2% of PBO-LLINs remaining in good condition at 18 months, the standard three-year replacement cycle appears inadequate. Additionally, PBO nets did not meet WHO-recommended efficacy thresholds against pyrethroid-resistant vectors, underscoring the need for improved net care and routine durability monitoring at 18 months to identify and replace damaged nets.

## Supporting information

S1 File SX checklist. (DOCX)

S2 File Combined net integrity PBO and LLINs-PLOS1. (XLSX)

S3 File Net bioefficacy data. (XLSX)

S4 File House structure and status. (XLSX)

S5 File PLOSOne_Human_Subjects_Research_Checklist-Other_upload. (DOCX)
